# ‘Hyperbinding’ in functional movement disorders: role of supplementary motor area efferent signalling

**DOI:** 10.1093/braincomms/fcae464

**Published:** 2025-02-24

**Authors:** Anirban Dutta

**Affiliations:** Centre for Systems Modelling and Quantitative Biomedicine, Department of Metabolism and Systems Science, College of Medicine and Health, University of Birmingham, Birmingham B15 2TT, UK

I wish to provide a constructive critique of the paper titled ‘Increased beta synchronization underlies perception-action hyperbinding in functional movement disorders (FMD)’ by Pastötter *et al*.,^1^ recently published in *Brain Communications*. My observations are intended to engage in scholarly discourse and further the understanding of the topic addressed in this important work. While the study contributes to the understanding of neural dynamics in FMD, I believe the paper overlooked a critical aspect of motor control: the role of the supplementary motor area (SMA) in sensorimotor integration by providing an efferent signal for sensory suppression^2^

The study’s principal finding—heightened beta synchronization observed during the post-movement stages—presents an intriguing contribution to the field. However, the interpretation of ‘hyperbinding’ as the primary mechanism underlying perception–action integration in patients with FMD may overlook a critical aspect of Bayesian sensorimotor integration principles.^3^ Research has demonstrated that SMA plays a crucial role in sending efferent signals to modulate sensory feedback during voluntary actions^2^ and the functional importance of cortical beta in the motor efferent command^4^ from feedforward estimation.^3^ Specifically, Haggard and Whitford’s study^2^ on sensory suppression argues that the SMA provides an efferent signal that contributes to reducing sensory input from self-generated movements. This mechanism ensures that sensory reafference is suppressed, aiding in distinguishing between self-generated and external stimuli for sensorimotor integration.^3^

In the context of FMD, patients often struggle to distinguish between voluntary and involuntary movements. This difficulty may stem from a failure in the efference mechanism, particularly involving the SMA.^4^ Moreover, elevated beta synchronization during the post-movement stages likely reflects a reduction in uncertainty associated with the brain’s feedforward estimations^3^ which, in turn, would lead to diminished Bayesian updating based on new sensory feedback according to Bayesian learning model^5^—a phenomenon referred to as ‘hyperbinding’ by Pastötter *et al*.^1^ The paper by Pastötter *et al*.^1^ discusses increased post-movement beta synchronization; however, it does not fully explore how this heightened beta activity may indicate dysfunction in the SMA’s efferent signalling and its role in sensorimotor integration.^3^ Such dysfunction in efferent signalling could provide a more comprehensive explanation for the ‘hyperbinding’ phenomenon observed in FMD. Understanding these mechanisms could pave the way for targeted interventions, as dysfunctional post-movement beta synchronization might be addressed through a combination of top-down motor imagery^6^ and bottom-up peripheral feedback^7^ for biofeedback training.^8^

## Data Availability

Data sharing is not applicable to this article since no new data were generated or analysed.
